# Neutrophils seeking new neighbors: radiotherapy affects the cellular framework and the spatial organization in a murine breast cancer model

**DOI:** 10.1007/s00262-024-03653-1

**Published:** 2024-03-02

**Authors:** C. M. Reichardt, M. Muñoz-Becerra, A. Rius Rigau, M. Rückert, R. Fietkau, G. Schett, U. S. Gaipl, B. Frey, L. E. Muñoz

**Affiliations:** 1 Department of Internal Medicine 3, Rheumatology and Immunology, Friedrich-Alexander-Universität Erlangen-Nürnberg (FAU) and Universitätsklinikum Erlangen, Ulmenweg 18, 91054 Erlangen, Germany; 2 Deutsches Zentrum Für Immuntherapie (DZI), Friedrich-Alexander-Universität Erlangen-Nürnberg (FAU) and Universitätsklinikum Erlangen, Erlangen, Germany; 3 Translational Radiobiology, Department of Radiation Oncology, Universitätsklinikum Erlangen, FAU Erlangen-Nürnberg, Erlangen, Germany; 4 Department of Radiation Oncology, Universitätsklinikum Erlangen, FAU Erlangen-Nürnberg, Erlangen, Germany; 5 Comprehensive Cancer Center Erlangen-EMN, Erlangen, Germany

**Keywords:** Cancer, Cellular neighborhood, Image mass cytometry, Neutrophil, Radiotherapy

## Abstract

Neutrophils are known to contribute in many aspects of tumor progression and metastasis. The presence of neutrophils or neutrophil-derived mediators in the tumor microenvironment has been associated with poor prognosis in several types of solid tumors. However, the effects of classical cancer treatments such as radiation therapy on neutrophils are poorly understood. Furthermore, the cellular composition and distribution of immune cells in the tumor is of increasing interest in cancer research and new imaging technologies allow to perform more complex spatial analyses within tumor tissues. Therefore, we aim to offer novel insight into intra-tumoral formation of cellular neighborhoods and communities in murine breast cancer. To address this question, we performed image mass cytometry on tumors of the TS/A breast cancer tumor model, performed spatial neighborhood analyses of the tumor microenvironment and quantified neutrophil-extracellular trap degradation products in serum of the mice. We show that irradiation with 2 × 8 Gy significantly alters the cellular composition and spatial organization in the tumor, especially regarding neutrophils and other cells of the myeloid lineage. Locally applied radiotherapy further affects neutrophils in a systemic manner by decreasing the serum neutrophil extracellular trap concentrations which correlates positively with survival. In addition, the intercellular cohesion is maintained due to radiotherapy as shown by E-Cadherin expression. Radiotherapy, therefore, might affect the epithelial–mesenchymal plasticity in tumors and thus prevent metastasis. Our findings underscore the growing importance of the spatial organization of the tumor microenvironment, particularly with respect to radiotherapy, and provide insight into potential mechanisms by which radiotherapy affects epithelial–mesenchymal plasticity and tumor metastasis.

## Introduction

Cancer is a widespread disease in most parts of the world. One of the most prevalent malignancies is breast cancer (BC) with 7.8 million women diagnosed within the past 5 years in 2020 (WHO). The disease can develop spontaneously, in rare cases in connection with pregnancy, childbirth or mutations [1]. In any case, the development of a tumor can be attributed to a three-stage failure of the immune system also referred to as immunoediting [2]. In healthy individuals, the immune system is able to detect and eliminate emerging and fully developed tumor cells. This protective system fails, if tumor cells gain characteristics by which they can evade the immune surveillance; they become invisible to the immune system and a clinically detectable disease is formed [3]. If the immune surveillance fails and a tumor develops, the immune system stays in contact with the malignancy but can be modulated by several factors [4]. Therefore, the molecular and cellular composition of the tumor microenvironment (TME) has been reported to exert great influence on the tumor immune response [5]. Immune cells can be recruited to the TME and polarized toward an immunosuppressive phenotype, thus promoting a pro-tumor immune response [6, 7]. T lymphocyte infiltration, for instance, can be hindered by the TME and infiltrating lymphocytes are driven to energy by altered ion concentrations and pH [8, 9]. Another very popular example is macrophages that can be polarized into M1 and M2 macrophages by the TME [10]. Whereas M1 macrophages are described as anti-tumor macrophages, the M2 phenotype is considered pro-tumor and associated with a bad prognosis [11, 12]. The polarization of macrophages has been shown to be inducible by irradiation. Dependent on the irradiation dose and scheme the cells are either polarized toward M1 or M2 [13, 14]. However, the identification of polarized macrophages in situ remains a major challenge [15].

Unlike other myeloid cells, such as macrophages, neutrophils have been largely underestimated in cancer research for many years although they account for up to 70% of circulating leucocytes in humans [16]. As part of the innate immune system, neutrophils react faster than cells of adaptive immunity and thus form the first line of defense against any pathological changes in the body [17]. They have unique defense strategies that distinguish them from other phagocytes. Most notable is the formation of neutrophil extracellular traps (NETs), which are strands of extracellular DNA (ecDNA) coupled to various neutrophil-related proteins such as neutrophil elastase [16]. In terms of the tumor immune response, NETs have been associated with E-Cadherin-loss and thus increased metastasis [18, 19]; other effector functions of neutrophils, such as the release of reactive oxygen species (ROS), have been described to support angiogenesis and tumor growth [20]. However, neutrophils are not solely described unfavorable regarding the anti-tumor immune response; they can counteract tumor growth by the release of anti-microbial mediators with cytotoxic properties and the recruitment of other immune cells [20]. Neutrophils have been described as a heterogeneous group with multiple attempts to define subgroups such as N1 and N2 neutrophils, similar to macrophages. However, there is increasing evidence that neutrophils cannot be categorized into subgroups, but rather must be considered as cells that move along a developmental gradient with multiple stages and characteristics [21, 22]. Regardless of whether and how neutrophils can be divided into subgroups, it has been demonstrated that these cells, like macrophages, can also develop pro- or anti-tumor properties due to soluble factors from the TME and also irradiation [23, 24]. Recent results from a machine learning-based immunophenotyping study by Hecht et al. underscore the importance of myeloid cells in cancer research, as their distribution in peripheral blood was the strongest predictor of treatment response in head and neck cancer patients treated with radio-chemotherapy [25].

However, in addition to the immune cellular composition of peripheral blood and TME, the spatial organization of tumors has recently received increasing attention, and tumors are increasingly viewed as organized structures rather than uniform heterogeneous cell masses. [26]. Research into the spatial architecture of tumors has gained attention in recent years, with milestones achieved in just a few years using high-throughput analysis and deep learning algorithms. However, there are still many unanswered questions, especially regarding the modification of the spatial organization of tumors by different treatment strategies [27].

Radiotherapy (RT), for instance, has been shown to affect the immune system and anti-tumor immune responses, although its effect on spatial organization within a tumor remains unclear. RT, besides its direct DNA damaging effects, acts in an immune stimulatory manner as an in situ tumor vaccine by killing tumor cells, which are then recognized by antigen presenting cells and presented to cells of the adaptive immune system [28]. However, RT can induce multiple types of cell death inducing either pro- or anti-inflammatory responses. The induction of apoptosis rather than necrosis, for example, maintains tumor-tolerance mediated by phagocytes [29]. However, the combination of the cell death type and the recruited immune cells varies and so does the effect on the immunogenicity of the tumor.

RT is an important, indispensable, part of modern BC treatment. Up to 70% of all patients receive this treatment, which reduces the risk of local recurrence of the tumor by up to 60% [30, 31]. In the light of this, our study aims to investigate the effects of irradiation on neutrophils in BC, focusing on their spatial organization within the TME as well as their effector functions and potential impact on metastasis.

## Material and methods

### Tumor mouse model

All animal experiments were approved by the “Regierung von Unterfranken” (approval number: 55.2.2–2532-2–1131) and performed in accordance with the guidelines of the Federation of European Laboratory Animal Science Associations (FELASA). Murine syngenic tumors were established in Balb/c mice by injection of 0.3 × 10^6^ TS/A breast cancer cells, which were kindly provided by Prof. Lollini (Bologna, Italy) and tested to be free of mycoplasma contamination, into the flank of the mice.

### Irradiation treatment

The tumors were locally irradiated with 2 × 8 Gy on day 12 and 15. The tumors were irradiated according to protocols for small animal partial body irradiation using a conventional clinical system with high quality control [32]. In brief, the mice were anesthetized with isoflurane and placed in a Plexiglas box and the anesthesia was maintained during whole irradiation procedure to prevent the movement of the mice. The irradiation was performed with a linear accelerator with 6MV photons and a 340° rotated gantry. The procedure was planned with computer tomography and dose was calculated similar to patient’s treatment. The delivered dose was confirmed by several measurements. The procedure is capable to hit the whole tumor and spare normal tissue as most as possible, as already published previously [33, 34]

### Tumor resection and preparation for image mass cytometry

The tumors were removed 7 days after the last irradiation treatment and subsequently transferred to ROTI®Histofix, 4% (Carl Roth, Karlsruhe) for 24 h. Afterward, samples were conserved in 70% ethanol (Carl Roth, Karlsruhe) until further proceedings. A 6 µm cast was excised from the growing edge of the tumor with a biopsy punch and embedded in paraffin. Slices of 5 µm were cut from the paraffin blocks and transferred to glass slides.

### Image mass cytometry staining

Paraffin slides were prepared by melting the paraffin at 62 °C for 2 h and rehydrated in a decreasing alcohol series for 5 min each. Slides were washed in ddH2O for 2 min and incubated in retrieval solution (10 mM Tri-natriumcitrate-dihydrate (Merck Millipore, Darmstadt), in ddH_2_O, pH 6.0) at 96 °C for 20 min. The slides and the retrieval solution were cooled down to room temperature and subsequently washed with water and PBS. The sections were blocked with blocking buffer (10% FCS (Thermo Fisher, Waltham), 2% BSA (Santa Cruz Biotechnology, Dallas)) in PBS for 1 h in a hydration chamber. Afterward, the blocking buffer was replaced with staining solution (lanthanide coupled antibodies (Table 1) in 0.5% BSA (Santa Cruz Biotechnology, Dallas) in PBS) and slides were incubated at 4° C overnight in a hydration chamber. Subsequently slides were washed two times in PBS with 0.2% Triton-X100 (Merck Millipore, Darmstadt) and two times in PBS. The sections were stained with Cell-ID™ Intercalator-Ir (Standard BioTools, #201192A) for 30 min at room temperature and washed with water. Air-dried slides were kept at 4 °C until scanning.

**Table 1:** List of IMC antibodies. Antibodies from Standard BioTools (former: Fluidigm) are pre-labeled. Antibodies from other companied were labeled using the Maxpar X8 Labeling Kit

Antigen	Metal conjugate	Final dilution	Company
Aquaporin 5	159 Tb	1:4000	Merk Millipore
Cytokeratin 5	149Sm	1:200	Abcam
Collagen Type I	169Tm	1:2400	Standard Biotools
E-Cadherin	158Gd	1:200	Standard Biotools
Cytokeratin 7	164Dy	1:400	Standard Biotools
Histone 3	176Yb	1:4800	Standard Biotools
Histone 3 (citrulline R2 + R8 + R17)	167Er	1:600	Abcam
CD4	156Gd	1:100	Standard Biotools
CD8a	162Dy	1:100	Standard Biotools
CD3	170Er	1:100	Standard Biotools
Neutrophil Elastase	165Ho	1:500	R&D
DNA	160Gd	1:50	Merk Millipore
CD11b	146Nd	1:200	Standard Biotools
Ly6G	166Er	1:200	Standard Biotools
Ki67	168Er	1:50	Standard Biotools
pS6	175Lu	1:200	Standard Biotools
SMAD4	155Gd	1:250	Abcam
TGF-β1	171Yb	1:4000	Novus-biologicals
Slug	150Nd	1:150	Novus-biologicals

### CyTOF acquisition

Data acquisition was performed on a Helios time-of-flight mass cytometer coupled to a Hyperion Imaging System (Standard BioTools). Laser ablation was performed at a frequency of 200 Hz. The device was calibrated, and a quality control was performed according to the manufacturer’s instructions. All IMC data were stored as.mcd and.txt files.

### Neutrophil elastase—DNA complexes ELISA

To determine the abundance of neutrophil elastase complexed with DNA (NE-DNA complexes) which are neutrophil-extracellular trap degradation products, a sandwich ELISA was performed (NE-DNA ELISA). Serum was collected from the animals at the day of death. 96-well plates (Thermo Fisher, Waltham) were coated overnight with 100 µl of an antibody against neutrophil elastase (R&D, #AF4517) 1:400 in coating buffer (0.1 M Na_2_CO_3_ (Merck Millipore, Darmstadt), 0.1 M NaHCO_3_ (Merck Millipore, Darmstadt), in ddH_2_O, pH: 9.6), washed three times with PBS-T (0.05% Tween® 20 (Merck Millipore, Darmstadt) in PBS) and subsequently blocked with 3% BSA (Santa Cruz Biotechnology, Dallas) in PBS for 2 h at room temperature while shaking. Afterward, the plate was washed three times with PBS-T, 40 µl serum was added per well, incubated at room temperature for 2 h while shaking and subsequently washed with PBS-T three times. 100 µl of the anti-DNA-POD antibody ((Sigma/Roche, #11544675001) 1:40 in incubation buffer (Sigma/Roche, #11544675001)) were added per well and incubated at room temperature for 90 min while shaking. Prior to adding 50 µl of TMB substrate solution (BioLegend, San Diego) the plate was again washed three times with PBS-T. To stop the reaction (after 30–60 min) 25 µl H_2_SO_4_ (Merck Millipore, Darmstadt) was added and the absorbance was measured at 450 nm (reference 620 nm).

### Data analysis

Fluidigm’s Hyperion imaging system was used to retrieve signals from 19 mass channels associated with biomarkers of interest in addition to two nuclear channels (Table 1). Multiplexed pseudo images were saved as *Mathcad* files and exported as individual *ome.tiff* images using the *MCD viewer* software. Tiff stacks were done with the *Image-J* software. Single cell masks were generated with the *Steinbock Docker Container* employing a deep learning-based image segmentation with the option *Mesmer* of *DeepCell*. The *imcRtools* package through the *read_steinbock* function summarized intensities per cell and channel and other properties of the segmented cells. This information was stored in a *SpatialExperiment* object, which was then used for graph construction, spatial visualization, and spatial analysis. We additionally used the package *Spectre* that defines a cell “0” outside of the previously defined cells and denoted this area as the intercellular space. We obtained the mean signal intensity for each marker and the cell “0”, which was used in addition to the *SpatialExperiment* object.

In order to visualize the location and interactions among cells, an *expansion interaction graph* based on the centroids of the cells was constructed with a *threshold* = *15* using the *buildSpatialGraph* function. The *cytomapper R* package provides the *cytomapperShiny* function that allows gating of cells based on their marker expression and visualization of selected cells directly on the images. This function allows to export gated cells in form of a *SingleCellExperiment* object per image. We identified myeloid cells (except neutrophils) and neutrophils based on the expression of CD11b and Ly6G and T cells with CD3, CD4 and CD8a, respectively. The *caret* package used the *SingleCellExperiment* object with the *trainControl* function containing the model training parameters and the *train* function. The model training assigned each cell to the class with highest probability. There were cases, where the highest probability was low and a cell could not be uniquely assigned to a class. We labeled these cells as Tumor cells. We used the *aggregateNeighbors* function of the *imcRtools R* package to define cellular neighborhoods. We computed the fraction of different cell types in the expansion interaction graph as constructed above and used *kmeans clustering* to group cells into 8 different cellular neighborhoods (CN).

### Statistics

Statistical analyses were performed with GraphPad—Prism 9. Statistical differences between two groups were calculated by an unpaired t test. Statistical differences between multiple groups were calculated using 2-way ANOVA. Correlation between two variables were calculated using Pearson correlation coefficient. Statistical significances in the survival were calculated using the Kaplan–Meier method. Significance values: *: *p* ≤ 0.05, **: *p* ≤ 0.01, ***: *p* ≤ 0.001.

## Results

**Fig. 1:**
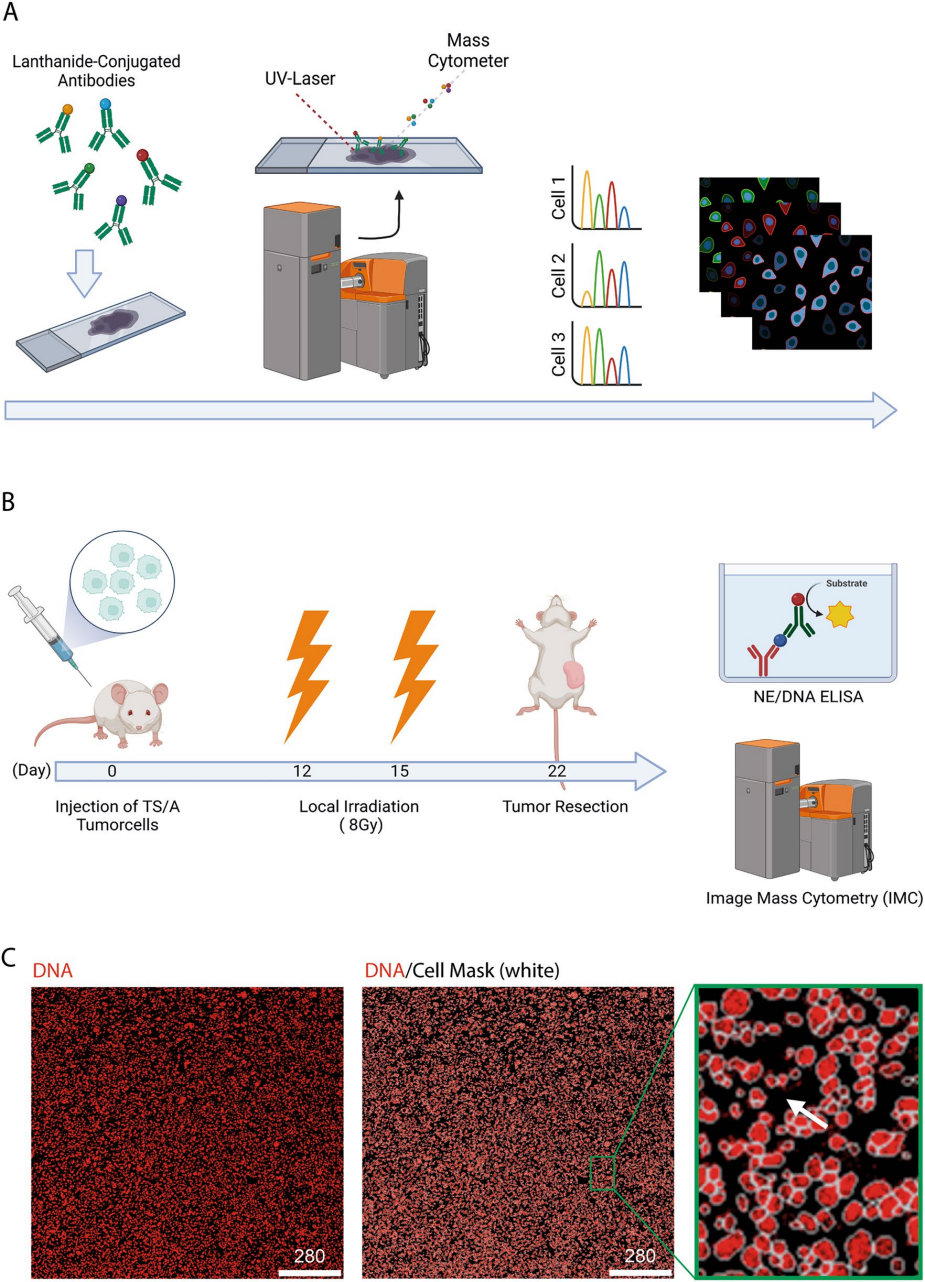
Experimental setup and analysis strategy. **A** IMC method. Tissue samples are stained with metal-conjugated antibodies and acquired by the Hyperion device. Therefore, a UV laser ablates the region of interest pixel by pixel and analyzes the material regarding the masses to distinguish the antibodies. As a result, a two-dimensional pseudo picture of each marker in its spatial arrangement in the tissue is created. Created with BioRender. **B** Experimental workflow. Created with BioRender. **C** IMC staining of DNA (red) in a breast carcinoma sample with cell mask (white). Close-up to cell mask with cellular DNA signal in red and membrane/background signal in black (white arrow)

Image mass cytometry (IMC) has been developed over the past 15 years and has become the forerunner method for performing spatial tissue analysis. IMC combines the advantages of immune fluorescence labeling and mass spectrometry allowing to determine the abundance of up to 40 denoted antigens within the structural frame of the tissue (Fig. 1a). In contrast to conventional immunofluorescence, CyTOF antibodies are conjugated to metal isotopes, overcoming the multiplexing limitations of conventional immunofluorescence and eliminating the major drawback of tissue- and treatment-specific autofluorescence in immunofluorescence staining. The UV laser beam built into the IMC system ablates the region of interest pixel by pixel (resolution: 1 µm^2^) and analyses the masses of the collected material to determine the abundance of each antibody per pixel by their masses. As the result pseudo images of the tissue are created to review each markers expression intensity in the spatial arrangement of the tissue. We used this novel technology to perform a multi-dimensional analysis of irradiated tumors (IT) compared to mock, non-treated tumors (NT) in a tumor model of murine breast cancer (Fig. 1b). Single cell information for each sample was obtained by cell masks defined by the *DeepCell* algorithm. However, antigens expressed predominantly on the cell membrane might be missed by this approach. Therefore, an additional mask was created to define the intercellular area of the tumor (Fig. 1c).

**Fig. 2:**
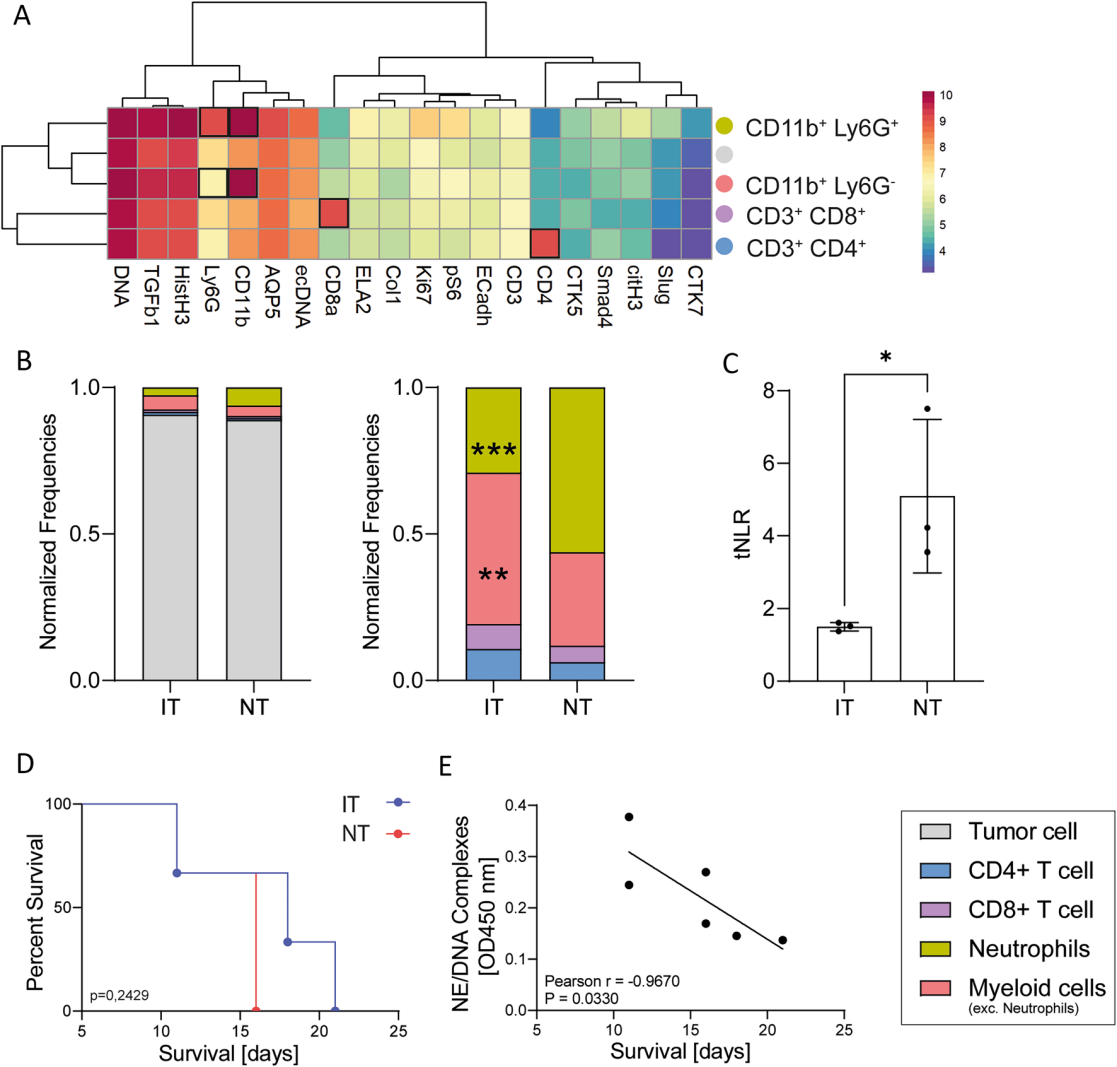
Neutrophils dominate the TME of untreated breast carcinomas. **A** Expression dependent differentiation of five cell types in breast carcinoma samples. **B** Normalized cell populations of breast carcinomas after irradiation therapy (IT) or without therapy (NT). The frequencies of all cell types were normalized based on the total cell count of the sample. **C** Ratio of neutrophils to lymphocytes in the analyzed tissue samples shown as tissue-NLR (tNLR). **D** Treatment dependent survival of tumor bearing mice. **e** Correlation of NE-DNA complexes and survival in serum of tumor bearing mice. Statistical differences between cell populations were calculated using a 2-way ANOVA. Statistical differences between tNLRs were calculated using an unpaired *t*-test. Correlation between two variables were calculated using Pearson correlation coefficient. Statistical significances in the survival were calculated using the Kaplan–Meier method. Significance values: *: *p* ≤ 0.05, **: *p* ≤ 0.01, ***: *p* ≤ 0.001. nNT = 3, nIT = 3

To determine the abundance of immune cells in the tumor, we used the function *cytomappershiny* from the package *cytomapper* of R. We selected neutrophils, CD4^+^ and CD8^+^ T-lymphocytes and myeloid cells (except neutrophils) manually based on the expression of specific markers (Fig. 2a). All unidentified cells were denoted as tumor cells. To compare samples with different cell numbers, the frequencies of all cell types were normalized based on the total cell count (Fig. 2b). We analyzed the abundance of these immune cell types in the tumor regarding the treatment applied (Fig. 2b) and found neutrophils to clearly dominate the compartment of infiltrating immune cells in the NT with significantly lower abundance in IT. On the contrary, cells of the myeloid lineage, which are not neutrophils, clearly dominate the compartment of infiltrating immune cells in the IT going along with a substantial higher abundance of T-cell in these tumors. Following the example of the neutrophil to lymphocyte ratio (NLR) commonly used in blood analyses, we aimed to determine the NLR in our tissue samples (Fig. 2c); hereinafter referred to as tissue-NLR (tNLR). The results are consistent with literature values of blood NLRs and show a significantly increase in NT. In contrast, the tNLR of IT samples remained within the normal, healthy range. Additionally, it was observed that tumor-bearing mice subjected to local irradiation treatment exhibited a higher survival rate compared to mice that did not receive any treatment (Fig. 2d). Since neutrophils appear to be affected by radiotherapy, we analyzed the effects of neutrophil effector functions on the survival of tumor-bearing mice. Therefore, we examined the presence of neutrophil elastase complexes with DNA (NE-DNA complexes) in serum, which are established markers of ongoing NET formation. Our analysis revealed a significant correlation between survival and the level of NE-DNA complexes in the serum of mice (Fig. 2e).

**Fig. 3:**
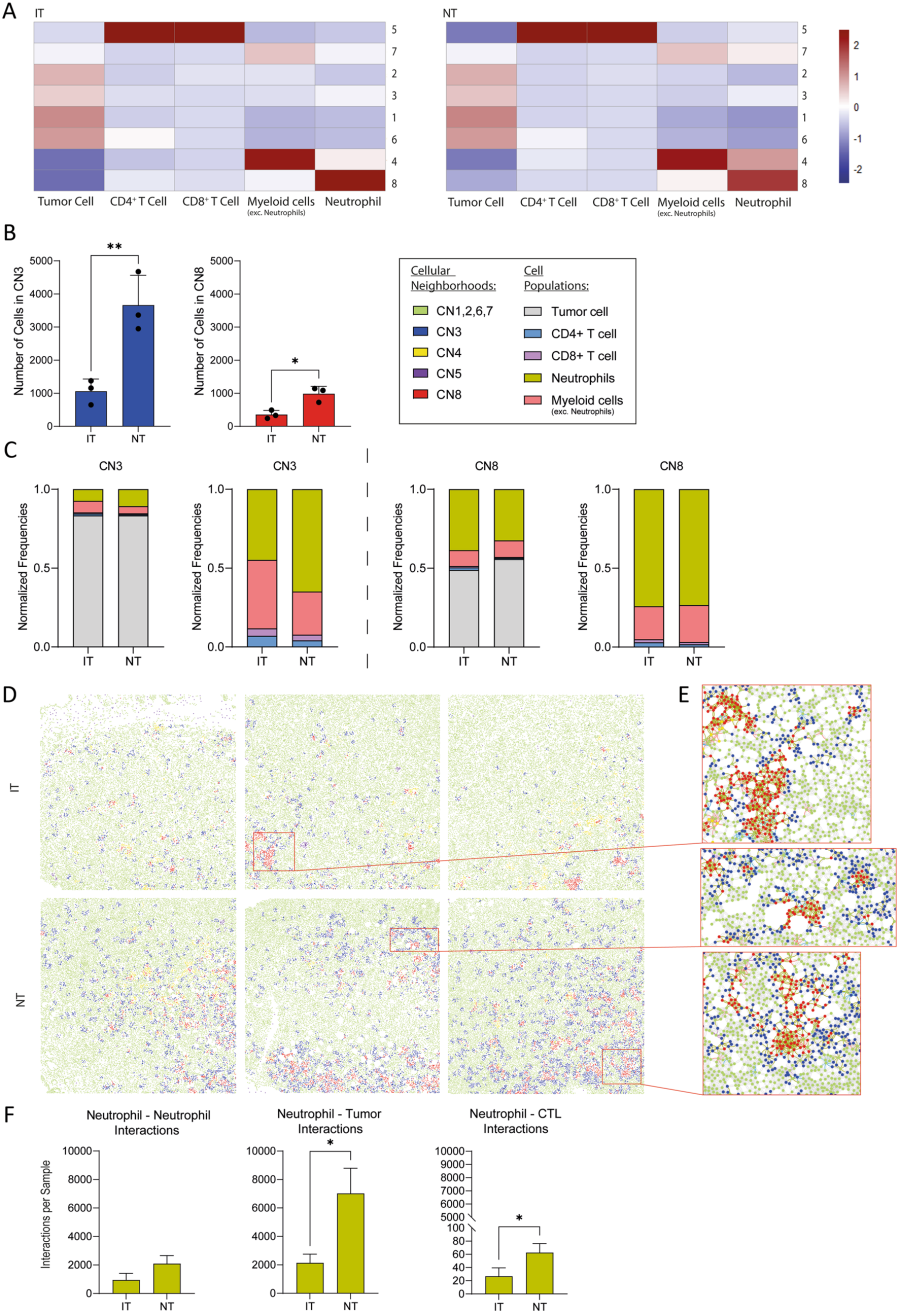
Neutrophil dominated cellular neighborhoods (CNs) are significantly decreased in breast carcinoma due to radiotherapy. **A** Heat map of the cellular composition of the eight defined CNs in treated and untreated tumors. **B** Number of cells dedicated to the CNs three and eight in IT and NT samples. **C** Normalized cellular and immune cellular composition of the CNs three and eight. The frequencies of all CNs were normalized based on the total cell count. **D** CN in IT and NT samples. **E** Close-up to intercellular interactions in tumor parts dedicated to the cellular neighborhoods three and eight. Cell dots are colored according to their CN, interaction lines are colored according to the cell type. **F** Interaction analyses between neutrophils and other neutrophils, tumor cells and cytotoxic T-lymphocytes (CTL). Statistical differences between CNs were calculated using a 2-way ANOVA. Statistical differences between two groups were calculated using an unpaired *t*-test. Significance values: *: *p* ≤ 0.05, **: *p* ≤ 0.01, ***: *p* ≤ 0.001. nNT = 3, nIT = 3

To further investigate the TME, we assessed the interactions among immune cells and tumor cells through spatial analysis in our breast carcinoma samples. Using the *buildspatialgraph* function of the *imcRtools R* package a spatial interaction graph (exemplary in Fig. 3e) of each sample is generated by calculating the 10 nearest neighbors of each cell in the two-dimensional space. Based on the spatial interaction graphs, the *aggregateNeighbors* function clusters the cells into eight CNs based on the kmeans clustering algorithm. Accordingly, CNs are groups of cells that cluster together based on their spatial localization within the tissue, their expression patterns and interaction frequencies with other cells. A CN is assigned to every individual cell within each sample. The relative abundance of each cell type within the eight neighborhoods is shown as a heat map of z values (Fig. 3a). CN five is dominated by T-lymphocytes; whereas, CN four and seven are dominated by cells of the myeloid lineage. CN one, two, three and six predominantly consist of tumor cells and CN eight is dominated by neutrophils.

When comparing the number of cells assigned to the eight CNs between the two treatment groups, significant differences could be detected for CN three and CN eight (Fig. 3b). To compare the frequencies of cell populations within the CNs in our samples of different cell counts, the frequencies of all CNs were normalized based on the total cell count (Fig. 3c). Having a closer look into the composition of the CN three and eight we found neutrophils to be highly represented regarding all cells of the neighborhood or the immune cell compartment respectively (Fig. 3a, c). Considering the spatial distribution of the CNs in the tumor sections, it seems that patches of cells denoted to CN eight (red) are surrounded by patches of cells denoted to CN three (blue) (Fig. 3d). Closer look into the tumor sections reveals that in CN eight are mostly neutrophil clusters that are surrounded by tumor cells belonging to CN three (Fig. 3e). Further analysis of the cell–cell interactions (Fig. 3f) reveals that NT have substantially higher neutrophils to neutrophil interactions and significantly more neutrophil to tumor cell interactions presumably resulting in the formation of the CN eight. Interestingly, we also found neutrophils to CD8^+^ cytotoxic T -lymphocyte interactions to be significantly increased in NT. The T -lymphocyte dominated CN five, however, is very low abundant in our samples and did not vary significantly between IT and NT.

**Fig. 4:**
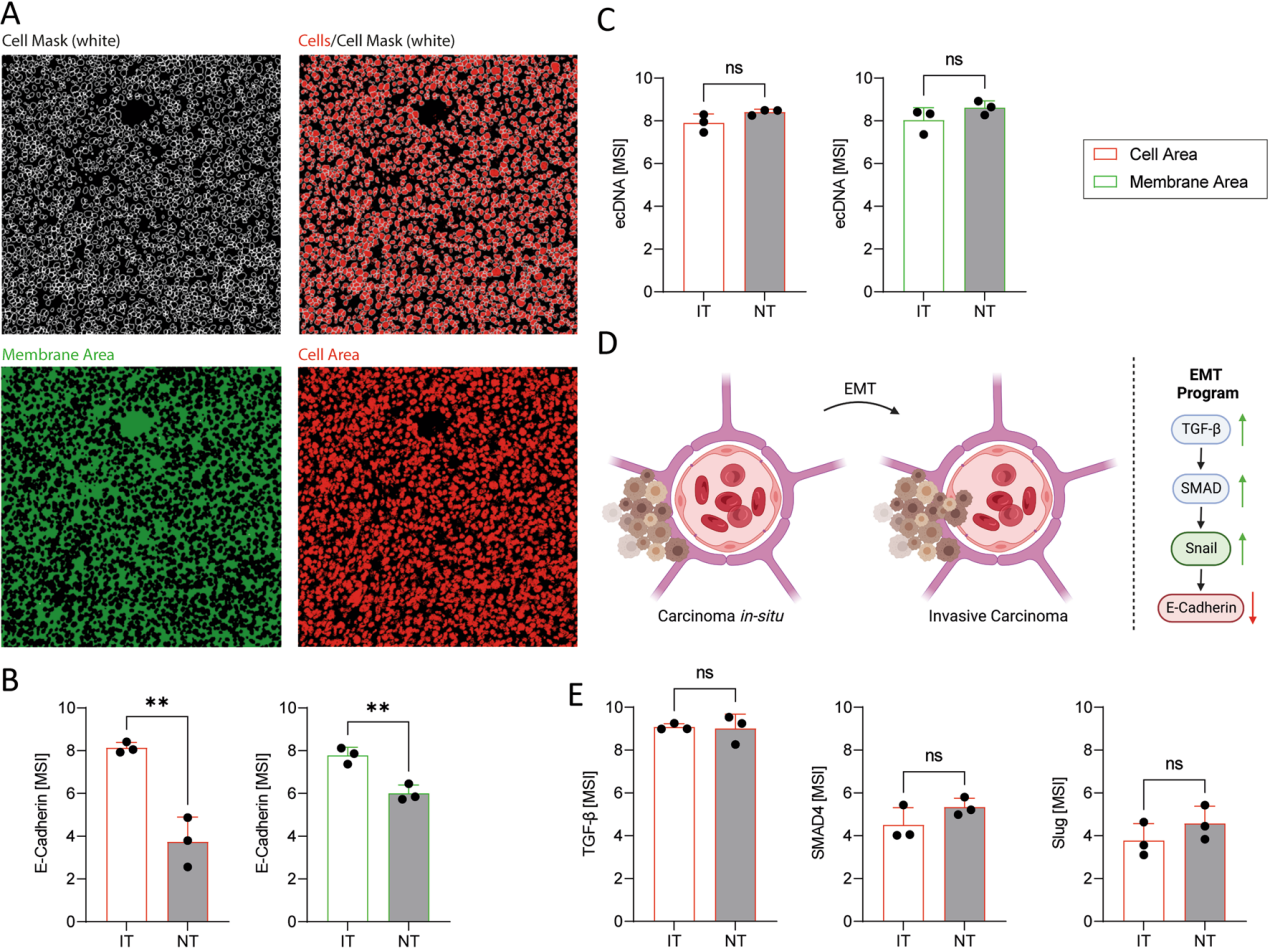
Radiotherapy prevents E-Cadherin loss in murine breast carcinoma. **A** Origin of signals from the cell and membrane/background area from breast carcinoma sections. **B** The mean signal intensity (MSI) of E-Cadherin is significantly decreased in the cell area and in the membrane area of NT. **C** MSI of ecDNA in the cell area and the membrane area. **D** Molecular pathway of epithelial to mesenchymal transition (EMT). Created with BioRender. **E** MSI of TGF-*β*, SMAD4 and Slug in the cell area. Statistical differences between two groups were calculated using an unpaired *t*-test. Significance values: *: *p* ≤ 0. 05, **: *p* ≤ 0.01, ***: *p* ≤ 0.001. nNT = 3, nIT = 3

Given the fact, that neutrophil infiltration and NET-release is associated with loss of E-Cadherin expression in the context of cancer, we wanted to investigate the effect of the altered immune cell infiltration in the treated and untreated samples on the expression of E-Cadherin and metastasis-associated proteins. We, indeed, found the intracellular expression (Fig. 4a) of the adherens junction protein E-Cadherin to vary between the treatment groups, with significantly reduced expression in NTs (Fig. 4b), that show significantly increased neutrophil infiltration (Fig. 2b). Furthermore, we analyzed the E-Cadherin signal in the membrane area (Fig. 4a) to exclude the possibility that our cell mask neglects parts of the membrane where E-Cadherin is expressed. Similar to the cell area, E-Cadherin expression is significantly decreased in the membrane area of neutrophil-rich NTs (Fig. 4b). Additionally, we analyzed ecDNA as a NET marker in the tissue samples and found it visibly but not significantly increased in neutrophil-rich NTs (Fig. 4c). Due to the association of E-Cadherin-loss and neutrophils as well as NET-release to epithelial to mesenchymal transition (EMT) -mediated metastasis (Fig. 4d) we aimed to analyze additional mediators that are known to contribute to the multidirectional epithelial–mesenchymal plasticity (EMP) in the tissue. Therefore, we found the expression of SMAD4 and Slug visibly, but not significantly, increased in tumors of mice that received no treatment; whereas, TGF-*β* showed no alteration between the treatment groups (Fig. 4e).

Through IMC and spatial neighborhood analyses of mouse mammary tumors, we have demonstrated that radiotherapy significantly reduces the abundance of neutrophils as well as the ratio of neutrophils and lymphocytes in the tissue and the occurrence of neutrophil-rich CNs. Moreover, we found evidence for the systemic impact of RT on neutrophils, leading to decreased NET concentrations in the serum, which exhibit a positive correlation with survival rates. Furthermore, our data demonstrate that radiotherapy treatment, going along with decreased neutrophil counts, support the expression of E-Cadherin potentially leading to an enhancement of intercellular cohesion with the potential to affect the multidirectional EMP in the tumors and the potential to prevent metastasis formation.

## Discussion

Neutrophils have been widely overlooked in cancer research for decades due to their short-lived nature. However, they have various effector functions that can either support or hinder tumor progression [35]. Their most prominent effector function is the release of NETs to which both pro- and anti- inflammatory properties are attributed [36]. Similar to what we have previously found in a melanoma model [37], we observed that RT significantly reduces the abundance of neutrophils while at the same time increases the proportion of other myeloid cells in the tumors of the current study. This was also associated with lower serum concentration of NET-markers correlating with survival of the tumor bearing mice. Regarding cancer, NETs are generally considered unfavorable as they promote tumor growth and progression as well as metastasis and tumor-associated thrombosis [20, 38]. Accordingly, increased serum NET-levels have been associated with poor disease-free survival in colorectal cancer and has been attributed to the pro-metastatic properties of NETs [39]. Furthermore, NETs have been demonstrated to enhance migratory ability of human BC cells in vitro by altering the mesenchymal phenotype of the cells [40] and to increase motility in colorectal cancer cells potentially by alterations in the expression of ZEB1, Slug and E-Cadherin [18]. Accordingly, we showed that IT with decreased neutrophil infiltration have an increased expression of E-Cadherin. Therefore, RT might affect epithelial–mesenchymal plasticity and thus prevent metastasis, possibly mediated by neutrophils.

Moreover, we found the tNLR to be reduced due to RT. The NLR is an established prognostic marker with great interest in cancer research and care [41]; it is usually determined by blood analyses, is a common prognostic marker applied in cancer, including BC, displaying the misbalance between cells of the innate and adaptive immunity [42, 43]. Relatively high neutrophil and low lymphocyte count result in high NLR values and are associated with poor survival outcome in various types of solid and non-solid tumors [44]. Although NLRs are commonly determined by blood tests there has been attempts to determine the NLR in tissue. In this regard, high tissue NLRs in esophageal squamous cell carcinoma [45] and non-small cell lung cancer [46], are associated with poor survival and high risk of relapse.

Most currently available prognostic markers and biomarkers, including the NLR, only consider cell abundance and their properties. However, an increasing, number of studies indicate that spatial organization of tumors has an at least similar information content with the potential to function as prognostic marker in head and neck cancer [26]. Although tumors are heterogeneous in terms of genetic alterations [47] and spatial organization the current understanding suggests that tumors have distinct landscapes that can be interpreted similar to maps [48]. Brain metastases from different tumors and primary brain tumors, for instance, can be distinguished based on the spatial organization of the TME [49]. Furthermore, IMC analyses together with deep learning algorithms are capable of predicting the survival of treatment-naïve lung adenocarcinoma patients [50]. Jackson et al. and Tietscher et al. have shown in recent years that image mass cytometry has the power to improve breast cancer care. They have shown that imaging data are more predictive of patient clinical outcome than single-cell data without spatial information, and their approaches offer novel marker combinations for tumor classification and thus treatment selection [51, 52].

Naturally, the question arises as to how such novel prognostic markers are affected by cancer treatments such as RT, and whether an appropriate treatment could be predicted for each patient. Although there is a lack of data regarding the effect of RT on the spatial organization of the tumor and the possibility to predict treatment outcome, the spatial organization of irradiated tumors has been studied. Based on these investigations, head and neck tumors that were treated with RT and immunotherapy show distinct spatial features that can be associated with the clinical disease outcome [26]. Our IMC data demonstrate that the abundance of neutrophils and neutrophil clusters, represented by neutrophil-rich neighborhoods, is increased in untreated tumors. Such aggregates of neutrophils have already been described prior to metastasis formation in a murine breast cancer model and are associated with a bad disease outcome [53]. In RT-treated tumors, however, other cells of the myeloid lineage than neutrophils dominate the TME which goes along with the findings of Bravatá et al. [54] who demonstrated that the expression of CCL2, a chemoattractant for monocytes and macrophages, is increased in tumors due to irradiation.

Neutrophils and other myeloid cells are considered to be heterogeneous with the potential to develop either pro- or anti-tumor phenotypes but the effect of radiotherapy in this regard remains contradictory. Therefore, Takeshima et al. demonstrated that neutrophil infiltration can lead to a ROS-induced sterile inflammation and tumor mass reduction [55]. Wisdom et al. [56] however, showed that decreased neutrophil counts are associated with an increased survival in patients with cervical cancer that receive radio-chemotherapy. Regarding macrophages, Leblond et al. [57] reported that pro-tumoral M2 macrophages are more resistant to RT that M1 macrophages in the context of glioblastoma. Macrophage polarization by irradiation, however, has also been described dependent on the irradiation dose and scheme [13]. In our model we cannot distinguish between pro- and anti- tumoral neutrophils or M1 and M2 macrophages. However, we found the absence of neutrophils associated with RT and generally desirable regarding the survival. A further investigation of the polarization of the myeloid cells in IT and NT could reveal the effect of RT in this regard. For neutrophils, however, it remains discussed if this short-lived cell population can be divided into subsets or if those cells develop along a gradient with varying function and polarization [21].

Furthermore, it must be considered that our approach does not cover all immune cells populations and B-cells or NK-cells, for example, are not displayed in the analyses. Furthermore, TS/A tumor cells express myeloid growth factors which might lead to an increased abundance of cells of the innate immunity. In addition, the hormonal and/or CXCR2 status of the tumors may affect the immune response, which was not considered in this study. Similarly, it must be considered that the genetic background of different inbreed mouse strains impact the composition of the immune system. Therefore, BALB/c mice have been demonstrated to have neutrophils of higher functional efficiency compared to, for example, CBA/CaH mice [58]. Accordingly, these limiting factors must be considered when looking at the results of the study. Other breast cancer models should be studied to confirm that these findings are not solely related to the intrinsic biology of the TS/A tumors and the genetic background of the mice.

Our findings, nevertheless, underscore the growing importance of considering both cellular composition and spatial distribution of immune cells within the tumor, particularly in the context of RT, and shed light on potential mechanisms that influence treatment outcomes and metastatic potential. Novel imaging techniques such as IMC could have the potential not only to predict treatment outcomes, but also to determine appropriate treatment for each individual based on new deep learning algorithms.

## Data Availability

All relevant data are contained within the article: The original contributions presented in the study are included in the article, further inquiries can be directed to the corresponding author.

## Abbreviations

BC: Breast cancer
CN: Cellular neighborhood
CT: Computed tomography
CyTOF: Cytometry by time of flight
ecDNA: Extracellular DNA
EMP: Epithelial–mesenchymal plasticity
EMT: Epithelial to mesenchymal transition
FELASA: Federation of European laboratory animal science associations
IMC: Image mass cytometry
IT: Irradiated tumors
NETs: Neutrophil extracellular traps
NLR: Neutrophil to lymphocyte ratio
NT: Non-treated tumors
MSI: Mean signal intensity
ROS: Reactive oxygen species
RT: Radiotherapy
TME: Tumor microenvironment
tNLR: Tissue-neutrophil to lymphocyte ratio
